# Uptake and continuation of HIV pre‐exposure prophylaxis among women of reproductive age in two health facilities in Kisumu County, Kenya

**DOI:** 10.1002/jia2.26069

**Published:** 2023-03-13

**Authors:** Mercelline Ogolla, Omoto Lennah Nyabiage, Paul Musingila, Susan Gachau, Theresa M. A. Odero, Elijah Odoyo‐June, Boniface Ochanda, Aoko Appolonia, Elizabeth Katiku, Rachael Joseph, Cirrilus Ogolla, Linda Otieno, Francesca Odhiambo, Hong‐Ha M. Truong

**Affiliations:** ^1^ Afya Bora Consortium Department of Global Health University of Washington Seattle Washington USA; ^2^ Division of Global HIV & TB (DGHT) Center for Global Health (CGH) US Centers for Disease Control and Prevention Kisumu Kenya; ^3^ Center for Microbiology Research Kenya Medical Research Institute Kisumu Kenya; ^4^ Department of Medicine University of California San Francisco San Francisco California USA

**Keywords:** adolescent girls and young women, continuation, HIV prevention, oral pre‐exposure prophylaxis, uptake, women of reproductive age

## Abstract

**Introduction:**

In 2020, Kenya had 19,000 new HIV infections among women aged 15+ years. Studies have shown sub‐optimal oral pre‐exposure prophylaxis (PrEP) use among sub‐populations of women. We assessed the uptake and continuation of oral PrEP among women 15–49 years in two health facilities in Kisumu County, Kenya.

**Methods:**

A retrospective cohort of 262 women aged 15–49 years, initiated into oral PrEP between 12 November 2019 and 31 March 2021, was identified from two health facilities in the urban setting of Kisumu County, Kenya. Data on baseline characteristics and oral PrEP continuation at months 1, 3 and 6 were abstracted from patient records and summarized using descriptive statistics. Missing data in the predictor variables were imputed within the joint modelling multiple imputation framework. Using logistic regression, we evaluated factors associated with the discontinuation of oral PrEP at month 1.

**Results:**

Of the 66,054 women screened, 320 (0.5%) were eligible and 262 (82%) were initiated on oral PrEP. Uptake was higher among women 25–29 years as compared to those 15–24 years (77% vs. 33%). Oral PrEP continuation declined significantly with increasing duration of follow‐up; 37% at month 1, 21% at month 3 and 12% at month 6 (*p*<0.05). In the adjusted analysis, women 15–24 years had lower adjusted odds of continuing at month 1 than women ≥25 years (adjusted odds ratio [aOR]: 0.41, 95% CI: 0.21–0.82). There was no association between being sero‐discordant and continuation of oral PrEP at month 1 (aOR; 1.21, 95% CI 0.59–2.50). Women from the sub‐county hospital were more likely to continue at month 1 of follow‐up compared to women enrolled in the county referral hospital (aOR 5.11; 95% CI 2.24–11.70).

**Conclusions:**

The low eligibility for oral PrEP observed among women 15–49 years in an urban setting with high HIV prevalence calls for a review of the screening process to validate the sensitivity of the screening tool and its proper application. The low uptake and continuation among adolescent girls and young women underscores the need to identify and address specific patient‐ and facility‐level barriers affecting different sub‐populations at risk for HIV acquisition.

## 1 INTRODUCTION

Globally, women and girls account for 53% of people living with human immunodeficiency virus (PLHIV). At the end of 2021, despite a 52% reduction in new HIV infections since the peak in 1997, women 15 years and above still accounted for 44% of 1.5 million newly infected people globally. In East and Central Africa, women and girls accounted for 58% of new HIV infections in 2021 [1, 2]. Strategies to reduce the risk of HIV infection among women in sub‐Saharan Africa aim to overcome the biological, behavioural and structural drivers of HIV transmission, including unequal cultural, political, social and economic status in society. Oral pre‐exposure prophylaxis (PrEP), the use of antiretroviral (ARV) drugs to prevent HIV acquisition by individuals with ongoing risk of HIV infection, has been adopted as a key biomedical prevention strategy in global HIV programmes, including Kenya. Unlike other HIV prevention strategies, such as male condoms, antiretroviral therapy (ART) by an HIV‐positive partner or voluntary male medical circumcision, which depends on male partner cooperation, PrEP may offer much‐needed self‐efficacy for women at risk for HIV infection [3].

Oral PrEP has been shown to effectively reduce the risk of HIV acquisition in clinical trials in diverse settings and populations [4]. Multiple studies have shown that oral PrEP can reduce the chances of HIV infection to near zero when taken consistently and correctly [5, 6, 7]. The efficacy of oral PrEP depends on high uptake and adherence, particularly among women, as higher concentrations of ARVs are required in the female genital tract to confer protection. Therefore, the current oral PrEP strategy for heterosexual women requires a daily pill [8].

Clients on oral PrEP require regular follow‐up visits to enable healthcare providers to assess ongoing HIV risk and the appropriateness of continuing oral PrEP, in addition to monitoring for drug toxicities and providing adherence support. Suboptimal adherence to oral PrEP has been linked to the acquisition of HIV among people receiving oral PrEP during clinical trials [9].

Kenya has one of the largest HIV epidemics globally, with women being 62% of the 1.4 million people (15+ years), living with HIV. In 2020, there were 19,000 new HIV infections among women aged 15 years and above [10]. In 2016, the national HIV control programme in Kenya adopted oral PrEP as part of HIV of combination prevention for people at substantial ongoing risk of HIV infection [11]. Although there has been significant progress in rolling out oral PrEP in Kenya, there is sub‐optimal oral PrEP use by women of reproductive age (WRA) (15–49 years) in high HIV prevalence settings. We assessed the uptake and continuation of oral PrEP among WRA in two high‐volume facilities in Kisumu County, western Kenya, where HIV prevalence (17.3%) is substantially higher than the national average (4.9%) [12].

## 2 METHODS

### 2.1 Study setting and design

This retrospective cohort analysis was conducted in two out of the five public health facilities (sites) supported by the University of California San Francisco and the Family AIDS Care and Education Services (UCSF‐FACES) programme under the U.S. President's Emergency Plan for AIDS Relief (PEPFAR) in Kisumu Central Constituency, Kisumu County, western Kenya. The sites, classified as county and sub‐county hospitals in the Kenyan health structure and located within the central business district of Kisumu County, were selected based on hospital delivery volume, healthcare provider to patient volume, at least 500 PLHIV receiving ART, the highest number of oral PrEP initiations and at least 500 HIV tests conducted per year.

The study population included all WRA who: (1) were determined to have a high ongoing risk for HIV infection and eligible for PrEP (in discordant relationships; identified as key population (female sex worker [FSW] and people who inject drugs); sexual partners of index clients reached by routine partner notification services [PNSs]; attending the maternal and child health [MCH] clinic, and seeking services in the outpatient [OPDs] or inpatient departments [IPDs]); (2) initiated on oral PrEP between November 2019 and March 2021; and (3) whose data were available in individual client files and facility registers relating to PrEP services.

#### 2.1.1 Routine oral PrEP procedures

HIV prevention, care and treatment services were provided within the comprehensive care clinics (CCC) or integrated into MCH clinics. Oral PrEP was provided as part of the standard package of HIV prevention services routinely offered to eligible clients accessing inpatient and outpatient services [11].

Screening for oral PrEP eligibility includes behavioural risk assessment using a standardized PrEP risk assessment tool (see Appendix S1). This was conducted at all service delivery points in the facility. Once considered eligible, providers linked clients to PrEP service delivery points where further assessment and HIV testing are conducted before oral PrEP initiation.

Once initiated, clients are required to return for a follow‐up visit after 1 month, and thereafter, every 3 months for the duration of oral PrEP use. Procedures at follow‐up visits include: (1) an HIV blood test to ensure that oral PrEP is not dispensed to persons who have acquired HIV and who require ART; (2) clinical assessments and adherence monitoring, adverse drug reaction/events monitoring, and laboratory assessments for creatinine clearance and hepatitis B; (3) risk‐reduction counselling to establish ongoing HIV risk and need for continued oral PrEP use; (4) testing for Sexually Transmitted Infections (STIs) in sexually active adults and adolescents with signs or symptoms; (5) adherence counselling and follow up; and (6) assessment of pregnancy intent, and pregnancy testing where applicable. Clients with ongoing HIV risk and who are willing to continue using oral PrEP are given a new 3‐month oral PrEP prescription [11].

### 2.2 Data collection

Using a standardized tool, we abstracted data from records in the client file and facility registers, between 6 April 2021 and 6 May 2021. The completeness of the data in the registers differed in the two facilities—54% in the sub‐county facility and 44% in the county referral hospital. Data on the number of clients screened for eligibility were obtained from the HIV testing services (HTS) register, OPD, IPD registers, and PNS register and counterchecked against the HTS monthly summaries. From the PrEP register, we obtained data on those initiated on PrEP. Data collected was as at the time of oral PrEP initiation and it included socio‐demographic variables (age, marital status), reasons for PrEP initiation (client type, sex with a partner of known HIV‐positive status, transactional sex, condomless sex, or inconsistent condom use, intravenous drug use, multiple sexual partners and sex with partners with unknown HIV status), family planning use and reproductive health information, and reasons for discontinuation. The outcomes of interest were uptake and continuation of oral PrEP up to 6 months after initiation. Uptake was defined as the proportion of eligible WRA who initiated oral PrEP. Oral PrEP continuation was defined as documentation of a renewed prescription for oral PrEP during follow‐up visits. WRA who did not return for follow‐up visits were considered to have discontinued oral PrEP use at the time of the visit.

### 2.3 Data analysis

We used descriptive statistics to summarize client baseline characteristics, including socio‐demographic information, HIV risk factors, uptake and continuation of oral PrEP at months 1, 3 and 6, and reasons for oral PrEP initiation/discontinuation. The level of completeness was also assessed for participant characteristics. Factors associated with the completion of follow‐up visits at month 1 were explored using a logistic regression model with a logit link. In this study, missing data occurred in four explanatory variables, namely: family planning, use of PrEP in preconception, antepartum and postpartum period, referral source and marital status. The proportion of missingness ranged between 5.3% (14/262) in referral sources and 43.8% (115/262) in family planning. In standard statistical software, complete case analysis is the default method of handling missing data. A major limitation of this approach is loss of precision in inferences due to loss of information and risk for biased parameter estimates especially when data are not missing completely at random. To mitigate the impact of missingness, we employed multiple imputation (MI), a missing data handling technique that repeatedly draws from a regression model and the observed data to create multiple completed data sets [13]. Assuming a missing at random (MAR) mechanism, we jointly imputed partially observed variables (i.e. marital status, referral source, family planning and PrEP use) 30 times within the joint modelling imputation framework using the *mitml* [14] package in R Version 4.1.2. All the partially observed variables were categorical and were, therefore, imputed using the latent normal approach [13]. Fully observed explanatory variables (i.e. age, participant type, period of initiation into PrEP care and facility type) and the outcome of interest were used as the predictor variables in the imputation model. A key assumption in the joint modelling imputation approach is that the data can be described by a multivariate normal distribution from which imputations for all partial variables are drawn jointly using a single statistical imputation model [13, 15].

Thereafter, each imputed data set was analysed using a logistic regression model and final parameter estimates were pooled according to Rubin's rules [16]. In particular, univariable models were fitted to obtain unadjusted odds ratios and the corresponding 95% confidence intervals (CIs) of factors associated with oral PrEP continuation. Statistically significant variables (global *p*‐value of less than 0.25) in the univariable models were considered for inclusion in the multivariable logistic regression model. A *p*‐value cut‐off point of 0.25 was in line with the purposeful selection of covariates strategy based on the Wald test. This is because the more traditional levels such as 0.05 can fail in identifying variables known to be important [17]. This model‐building strategy was used under complete case analysis and after MIs as appropriate. Adjusted odds ratios (aOR) and their corresponding 95% CIs were used to measure the magnitude and direction of the association. Univariable and multivariable models were also fitted to complete case records and results compared to those obtained after MIs. All statistical tests were done at a 5% level of significance.

### 2.4 Ethical considerations

This study received ethical approval from the U.S. Centers for Disease Control and Prevention (CDC) Kenya, UCSF and the Kenya Medical Research Institute (KEMRI). It was also reviewed by the U.S. CDC human research protection procedures. CDC investigators did not interact with human subjects or have access to identifiable data or specimens for research purposes. A waiver of informed consent was obtained.

## 3 RESULTS

### 3.1 Background characteristics

A total of 66,054 WRA were screened for oral PrEP eligibility during the study period. Of these, 320 (0.5%) were eligible, and 262 (82%) were initiated on oral PrEP. Uptake was higher among WRA 25–29 years than those 15–24 years (67% vs. 33%). Characteristics of the 262 women initiated on oral PrEP are shown in Table 1. The majority 175 (67%) were aged 25–49 years, in a monogamous 145 (55%) or polygamous 41 (16%) marriage and referred from OPD 113 (43%) or voluntary testing and counselling clinics 83 (32%). Women in a discordant relationship represented 82 (31%) of those initiated on oral PrEP. A low proportion of 35 (13%) was either trying to conceive, pregnant or breastfeeding. The overall oral PrEP continuation rate was 37% at month 1, 21% at month 3 and 12% at month 6 of follow‐up. In month 1 of follow‐up, continuation was higher (41%) among women 25–49 years as compared to adolescent girls and young women (AGYW) 15–24 years (28%) but declined steeply to 12% and 13%, respectively, by month 6.

**Table 1:** Characteristics of women initiated on oral PrEP and continuation at months 1, 3 and 6 after initiation—Kisumu County 2019–2021

Characteristic	Initiated *n* (col %)	Continuation at 1 month n (row %)	Continuation at 3 months n (row %)	Continuation at 6 months n (row %)
**Eligible for PrEP (*N*=320)**				
**Age in years (*N*=262)**				
15–24	33% (87/262)	28% (24/87)	21% (18/87)	13% (11/87)
25–49	67% (175/262)	41% (72/175)	21% (37/175)	12% (21/175)
*Total*	262	37% (96/262)	21% (55/262)	12% (32/262)
**Marital status (*N*=244)**				
Single	10% (27/262)	30% (8/27)	11% (3/27)	7% (2/27)
Cohabiting	8% (21/262)	19% (4/21)	5% (1/21)	5% (1/21)
Married monogamous	55% (145/262)	41% (59/145)	28% (40/145)	18% (26/145)
Married polygamous	16% (41/262)	37% (15/41)	20% (8/41)	5% (2/41)
Separated/Divorced/Widowed	4% (10/262)	50% (5/10)	20% (2/10)	0
**Referral source (*N*=248)**				
Voluntary counselling and testing (VCT) site	32% (83/262)	25% (21/83)	14% (12/83)	8% (7/83)
Outpatient department	43% (113/262)	48% (54/113)	27% (31/113)	18% (20/113)
Maternal child health clinic	7% (19/262)	47% (9/19)	32% (6/19)	16% (3/19)
Other (programmes)	8% (21/262)	33% (7/21)	14% (3/21)	10% (2/21)
Partner notification services	5% (12/262)	0	0	0
**Client type (*N*=262)**				
Discordant couple	31% (82/262)	46% (38/82)	28% (23/82)	18% (15/82)
Others^a^	69% (180/262)	32% (58/180)	18% (32/180)	9% (17/180)
**Trying to conceive, pregnant or breastfeeding (*N*=160)**				
Yes	13% (35/262)	51% (18/35)	34% (12/35)	20% (7/35)
No	48% (125/262)	34% (42/125)	19% (24/125)	15% (19/125)
**Using family planning (*N*=147)**				
Yes	26% (67/262)	36% (24/67)	21% (14/67)	19% (13/67)
No	31% (80/262)	41% (33/80)	25% (20/80)	16% (13/80)
**Period of initiation into PrEP care (*N*=261)**				
Quarter 1 (Nov–Dec 2019)	9	22% (2/9)	22% (2/9)	22% (2/9)
Quarter 2 (Jan–Mar 2020)	50	56% (28/50)	38% (19/50)	24% (12/50)
Quarter 3 (Apr–Jun 2020)	32	44% (14/32)	22% (7/32)	13% (4/32)
Quarter 4 (Jul–Sep 2020)	71	42% (30/71)	20% (14/71)	13% (9/71)
Quarter 5 (Oct–Dec 2020)	49	31% (15/49)	20% (10/49)	10% (5/49)
Quarter 6 (Jan–Mar 2021)	50	14% (7/50)	4% (2/50)	0 (0)
**Facility type (*N*=262)**				
County referral hospital	82	17% (14/82)	5% (4/82)	0 (0)
Sub‐county hospital	180	46% (82/180)	28% (51/180)	18% (33/180)

^a^ Others—general population and key population.

### 3.2 Reasons for oral PrEP initiation

The most common documented reason for oral PrEP initiation was having an HIV‐positive sexual partner (56%). At month 1, the continuation rate was higher (36%) among WRA with single reasons for initiation as compared to those (23%) with multiple reasons. This is shown in Table 2.

**Table 2:** Reasons for initiating oral PrEP and continuation by reason for initiation—Kisumu County 2019–2021

*n* (%)	Initiated *n* (%)	Continuation at month 1 *n* (%)	Continuation at month 3 *n* (%)	Continuation at month 6 *n* (%)
**Reasons for initiating PrEP**				
Sexual partner of known HIV‐positive status	148 (56)	56 (38)	36 (24)	23 (16)
Engaging in transactional sex	1 (0)	0	0	0
Inconsistent or no condom use	14 (5)	9 (64)	6 (43)	3 (21)
Injection drug use with shared needles or syringes	1 (0)	1 (100)	0	0
Sero‐discordant couples trying to conceive	8 (3)	2 (25)	1 (13)	1 (13)
Having multiple sexual partners	6 (2)	0	0	0
Sex partners at high risk for HIV and HIV status unknown	54 (21)	15 (28)	9 (17)	4 (7)
Others (Epileptic)	1 (0)			
Missing	2 (1)	1 (50)	1 (50)	0
**Total—single reason for initiating PrEP**	235 (90)	84 (36)	53 (23)	31 (13)
**Multiple reasons for initiating PrEP (two or more reasons)**	27 (10)	11 (23)	3 (11)	3 (11)

### 3.3 Factors associated with oral PrEP continuation from enrolment to month 1 of follow‐up

In univariate logistic regression analysis, differences by age at enrolment, participant type, participant referral source, period and facility were significantly associated with oral PrEP continuation at month 1. Only age, client type, referral source and facility remained significant in the adjusted analysis. Generally, the magnitude and direction of associations were consistent between complete case analysis and after MIs of missing data variables. Cumulatively, 41.6% (109/262) of the total observations were discarded under complete case analysis. This loss of information led to parameter estimates with wider 95% confidence intervals compared to those estimated after MIs. After MIs, women 15–24 years had lower adjusted odds of oral PrEP continuation at month 1 than women ≥25 years (aOR: 0.41, 95% CI: 0.21–0.82). Further results showed that women enrolled in quarters 2, 3 and 4 did not have a different frequency of continuation relative to women enrolled in quarter 1. On the other hand, women enrolled in quarters 5 and 6 were significantly less likely to continue oral PrEP after month 1 (aOR; 0.25, 95% CI; 0.07–0.89, aOR: 0.20, 95% CI; 0.05–0.75, respectively) as compared to those enrolled in quarter 1. Finally, women who initiated oral PrEP at the sub‐county site were more likely to continue oral PrEP after month 1 compared to those in the county site (aOR 5.11; 95% CI 2.24–11.70). This is shown in Table 3.

**Table 3:** Factors associated with continuation of oral PrEP at month 1 after initiation—Kisumu County, 2019–2021

			Complete case analysis				Imputed datasets			
	Total	Continuation 1 month *n* (%)	Unadjusted OR (95% CI)	*p*‐value	Adjusted OR^a^ (95% CI)	*p*‐value	Unadjusted OR (95% CI)	*p*‐value	Adjusted OR (95% CI)	*p*‐value
	*n*=262	*n*=96								
**Age (years) (*N*=262)**										
15–24	87	24 (27.6)	0.54 (0.31–0.95)	0.03	0.46 (0.18–1.14)	0.03	0.54 (0.31, 0.95)	0.03	0.41 (0.21, 0.82)	0.01
≥25	175	72 (41.1)	1		1		1		1	
**Participant type (*N*=262)**										
General population	180	58 (32.2)	1	0.029	1	0.14	1	0.02	1	0.60
Discordant couple	82	38 (46.3)	1.82 (1.06–3.10)		1.2 (0.48–3.13)		1.82 (1.06, 3.10)		1.21 (0.59, 2.50)	
**Marital status (*N*=244)**										
Single	27	8 (29.6)	0.61 (0.25–1.49)	0.24			0.59 (0.24, 1.44)	0.31		
Cohabiting	21	4 (19)	0.34 (0.11–1.07)				0.35 (0.11, 1.10)			
Married monogamous	145	59 (40.7)	1				1			
Married polygamous	41	15 (36.6)	0.84 (0.41–1.72)				0.88 (0.43, 1.82)			
Separated/Divorced/Widowed	10	5 (50)	1.46 (0.40–5.46)				1.49 (0.40, 5.55)			
**Referral source (*N*=248)**										
Voluntary counselling and testing	83	21 (25.3)	1	<0.001	1	0.12	1	0.03	1	0.74
Outpatient department	113	54 (47.8)	2.70 (1.46–5.01)		1.31 (0.43–3.98)		2.76 (1.49, 5.11)		1.69 (0.74, 3.85)	
Maternal child health clinic	19	9 (47.4)	2.66 (0.94–7.45)		1.77 (0.37–8.54)		2.74 (0.98, 7.65)		1.12 (0.32, 3.95)	
Partner notification services	12	0 (0)			0 (0, 0)		0 (0, 0)		0 (0, 0)	
Other programmes	21	7 (33.3)	1.48 (0.53–4.15)		1.62 (0.23–1.24)		1.57 (0.56, 4.4.41)		1.17 (0.33, 4.08)	
**Use of PrEP in preconception, antepartum and postpartum period (*N*=160)**										
Yes	35	18 (51.4)	2.9 (0.98–4.47)	0.06	2.87 (0.96, 9.01)	0.06	2.09 (1.04, 4.24)	0.04	2.94 (0.98, 8.82)	0.06
No	125	42 (33.6)	1		1		1		1	
**Family planning use (*N*=147)**										
Yes	67	24 (35.8)	0.79 (0.41–1.55)	0.501			0.79 (0.41, 1.55)	0.5		
No	80	33 (41.3)	1				1			
**Period of initiation into PrEP care (*N*=261)**										
Quarter 1 (Nov–Dec 2019)	9	2 (22)	1	<0.01	1	0.04	1	0.002	1	0.02
Quarter 2 (Jan–Mar 2020)	50	28 (56)	0.73 (0.26, 2.01)		0.42 (0.08, 1.96)		0.74 (0.27, 2.02)		0.81 (0.26, 2.54)	
Quarter 3 (Apr–Jun 2020)	32	14 (44)	0.92 (0.34, 2.44)		0.33 (0.06, 1.48)		0.93 (0.35, 2.47)		0.96 (0.31, 3.00)	
Quarter 4 (Jul–Sep 2020)	71	30 (42)	0.47 (0.17, 1.24)		0.29 (0.06, 1.28)		0.48 (0.18, 1.26)		0.73 (0.23, 2.25)	
Quarter 5 (Oct–Dec 2020)	49	15 (31)	0.26 (0.09, 0.74)		0.06 (0.01, 0.46)		0.27 (0.07, 0.77)		0.25 (0.07, 0.89)	
Quarter 6 (Jan–Mar 2021)	50	7 (14)	0.16 (0.05, 0.50)		0.13 (0.02, 0.70)		0.17 (0.05, 0.52)		0.20 (0.05, 0.74)	
**Facility type (*N*=262)**										
County referral hospital	82	14 (17.1)		<0.001		<0.001		<0.001		<0.001
Sub‐county hospital	180	82 (45.6)	4.06 (2.18, 8.01)		6.75 (2.34, 21.18)		4.06 (2.13, 7.75)		5.11 (2.24, 11.70)	

Note: Missing data points for variables: Marital status *n*=28; Referral source *n*=14; Use of PrEP in preconception, antepartum and postpartum period *n*=102; Family planning use *n*=115; Period of initiation *n*=1.

^a^Adjusted OR (95% CI): Under complete case analysis, estimation of the parameter was based on 58.4 (153/262) of the total observations.

### 3.4 Reasons for oral PrEP discontinuation

Data on reasons for oral PrEP discontinuation were available for 33 (13%) of the women included in the analysis. Reasons included non‐adherence/poor adherence 21 (64%), undetectable viral load in index client 1 (3%) and loss to follow‐up 11 (33%).

## 4 DISCUSSION

Eligibility for oral PrEP among WRA in public health facilities in Kisumu County was low (0.5%) given the high prevalence of HIV (17.3%) in the general population [12]. This could be attributed to a combination of episodic rather than continuous engagement in risky behaviours, the mismatch between client HIV risk perception and actual risk, and suboptimal oral PrEP eligibility screening, including non‐disclosure of risky sexual behaviours. While possible reasons for lower‐than‐expected eligibility for PrEP in high prevalence settings include service uptake by a self‐selected low‐risk group, sub‐optimal sensitivity of the screening tool and improper application of the screening tool by providers, the available data and design of this study did not allow for further analysis to explore these possibilities. A study of perceptions of and interest in HIV PrEP use among AGYW in Lilongwe, Malawi found that the interest in using oral PrEP by AGYW was grounded in the perception of the severity of HIV infection and the desire for protection against HIV [18]. The Sustainable East Africa Research in Community Health (SEARCH) study in rural Kenya and Uganda found that while AGYW have among the highest HIV risk, they were less likely than women aged 25 years or older to initiate PrEP [19].

Despite the relatively low eligibility, overall uptake was high (82%), consistent with recently conducted oral PrEP demonstration projects in Benin and South Africa which found uptake of 87% and 98%, respectively [20, 21]. Of the 320 eligible WRA, 18% did not initiate PrEP. While the willingness to start PrEP, a critical consideration for initiation is a contributing factor, other possible reasons include perception of ongoing HIV risk, interdepartmental referrals where screening for eligibility is done at all entry points within the facility, but clinical evaluation and initiation done at the PrEP room located within the CCC; the healthcare workers’ strike in Kisumu County from November 2020 through to January 2021 with a spillover effect to February 2021 or the COVID‐19 pandemic in March 2020, which resulted in the prioritization of services, thus affecting PrEP services among others.

Oral PrEP uptake and continuation are driven by many factors. The most common reason for initiating oral PrEP was having a partner with known HIV‐positive status or unknown HIV status. While uptake was higher (89%) among WRA with a single reason for initiating PrEP as compared to those with multiple reasons (11%), there was no difference in the trend in continuation at months 1, 3 and 6. An ongoing challenge for many individuals is the fluctuating HIV risk perception over time. Risk indicators most closely associated with oral PrEP interest include behavioural and partner factors. For example, women with a known HIV‐positive sexual partner may stop taking oral PrEP if the relationship ends. Similarly, those in a stable monogamous relationship with an HIV‐positive partner may feel less compelled to continue oral PrEP over time if their partner has had a sustained undetectable viral load. Non‐adherence was the most commonly cited reason for discontinuation of oral PrEP. Improved patient follow‐up and documentation could provide further insights into the high attrition observed in this population.

Oral PrEP continuation declined significantly with increasing follow‐up duration; 37% at month 1, 21% at month 3 and 12% at month 6 of follow‐up (*p*<0.05). The low continuation rates were consistent with another study in Kenya among FSWs, which found the retention rate at 1, 3 and 6 months of follow‐up was 40.3%, 26.3% and 14.0%, respectively [22]. Similarly, other studies regarding FSWs in Benin, West Africa and South Africa also noted low retention rates of 58.6% and 22%, respectively, at 12 months of follow‐up [20, 21]. Conversely, a study in Senegal demonstrated high retention of 79.9% and 73.4% at 6 and 12 months, respectively, among FSWs [23]. Possible reasons for low PrEP continuation include decreased perceived risk (e.g. hiatus or end of a sexual relationship or partner known to be living with HIV became virally suppressed), side effects, daily pill burden, preference for condoms, partner's insistence and facility‐level factors, such as stigma‐related discomforts with accessing PrEP at CCCs, inconvenient clinic location or operating hours, long wait times and short refill dates [24]. Further research may provide insight into methods of ensuring PrEP readiness before initiation and reasons for early and later discontinuation.

Oral PrEP continuation among AGYW was significantly lower (28%) at month 1, compared to women 25–49 years (41%). This finding is consistent with the Prevention Options for Women Evaluation Research (POWER) study conducted in Kenya and South Africa, which found early drop‐off rates in the first few months after oral PrEP start of approximately 50%, with about 20% of AGYW restarting oral PrEP within 6 months [25]. The study in Malawi also found that about 70% of those initiated on oral PrEP were lost by the end of the first month [18]. AGYW is a priority population for HIV prevention. Despite PrEP initiation being motivated by high perceived HIV risk, adherence was lowest among women 15–24 years of age [19]. An analysis of the associations of fundamental social determinants of health known to be relevant to adolescent health with important adolescent‐health outcomes, including mortality, sexual health, health behaviours and mental health, found that family norms and attitudes, and social connections strongly affect adolescents’ behaviours [26]. Accordingly, there is a need to identify specific facilitators and barriers to the uptake and continuation of oral PrEP among AGYW to maximize the impact on HIV infections in this vulnerable sub‐population.

The enrolment period significantly affected oral PrEP continuation at month 1 (*p*‐value <0.05). There were no significant differences in oral PrEP continuation at month 1 among women enrolled in quarters 2–4 (Jan–Sep 2020) and those enrolled in quarter 1 (Nov–Dec 2019). However, women initiated into oral PrEP in quarters 5 and 6 (Oct 2020–Mar 2021) were highly (75–80%) less likely to continue oral PrEP after 1 month than those initiated on oral PrEP in quarter 1. This may be attributed to the healthcare workers’ strike in Kisumu County that began in Nov 2020 through to January 2021 with a spillover effect to February 2021. Mitigation measures and re‐prioritization of health services and resources following the onset of the COVID‐19 pandemic in March 2020 may have deterred healthcare‐seeking behaviours and reduced facility‐level capacity to effectively provide PrEP services, among others. Women initiated on oral PrEP from the sub‐county facility were more likely to continue at month 1 than women enrolled in the county facility. Further research will help to understand the differences in practice in different healthcare facilities leading to differences in oral PrEP retention rates and to standardize care.

### 4.1 Study limitations

This study has several limitations. First, the study relied on a review of medical records, introducing the possibility of reporting bias. Information on other key demographic characteristics, such as socio‐economic status and educational level, was unavailable. Missing data on renewed prescriptions were considered to be discontinuation. However, it was not possible to quantify the accuracy of this assumption and appropriate discontinuation due to reduced risk. In this study, we had several covariates with a substantial proportion of missingness. Our decision to perform MI to address missingness was guided by findings from recent studies, which have pointed out that unbiased results can be obtained even with large proportions of missing data (> 40%), provided the imputation model is properly specified and data are MAR. Moreover, the fraction of missing information is proposed as a better guide to the efficiency gains from MI than the proportion of missing data [27]. In our MI, we assumed that data were MAR approach, and therefore, sensitivity analyses are recommended to explore the robustness of the inferences to these assumptions.

## 5 CONCLUSIONS

This study uniquely contributes to the limited literature base on oral PrEP uptake and retention among WRA accessing care at public health facilities in the setting of a generalized HIV epidemic. The low eligibility for oral PrEP observed among women 15–49 years in a setting with high HIV prevalence calls for a review of the screening process to validate the sensitivity of the screening tool and its proper application. The low PrEP uptake and continuation among AGYW underscores the need to identify and address specific patient‐ and facility‐level barriers affecting different sub‐populations at risk for HIV acquisition. Longitudinal HIV risk monitoring for clients on oral PrEP with consistent documentation of outcomes and reasons for discontinuation could explicate and contextualize the high observed attrition and potential barriers to retention, and improve programming. Further research is important to understand the differences in practice associated with differences in retention rates at different levels of healthcare, and how PrEP continuation changes over the course of time as facilities grow accustomed to offering it and more providers have experience prescribing it.

## COMPETING INTERESTS

The authors declare no potential competing interests concerning the research, authorship and/or publication of this article.

## AUTHORS’ CONTRIBUTIONS

MO, OLN, BO and TMAO were responsible for the conception of the study. MO, AA, FO and RJ were responsible for the study's design. MO was responsible for the drafting of the original draft. MO, LO and CO were responsible for the acquisition of data. MO, SG, PM and RJ performed the data analysis. EOJ, EK, AA, OLN and HHMT were responsible for critically editing the manuscript for important intellectual content. All authors reviewed, read and approved the final version of the manuscript.

## FUNDING

This work has been supported in part by the U.S. President's Emergency Plan for AIDS Relief (PEPFAR) through the U.S. Centers for Disease Control and Prevention (CDC), Division of Global HIV&TB (DGHT) under the terms of Cooperative Agreement NU2GGH001947 to the University of California San Francisco‐UCSF Clinical.

## DISCLAIMER

The findings and conclusions in this report are those of the authors and do not necessarily represent the official position of the funding agencies.

## Supporting information


**Appendix S1**: PrEP Rapid Assessment Screening Tool.

## Data Availability

This data is owned and protected from release by the laws and regulations of Kenya. De‐identified data set will be made accessible through a public domain using a URL or repository access from the Ministry of Health website after Septemebr 2026.
