# Supplementary figures and images for: Uptake and continuation of HIV pre‐exposure prophylaxis among women of reproductive age in two health facilities in Kisumu County, Kenya
Source: J Int AIDS Soc. 2023 Mar 13;26(3):e26069. doi: 10.1002/jia2.26069 (PMC10009800; doi:10.1002/jia2.26069)

Appendix 1: PrEP Rapid Assessment Screening Tool.


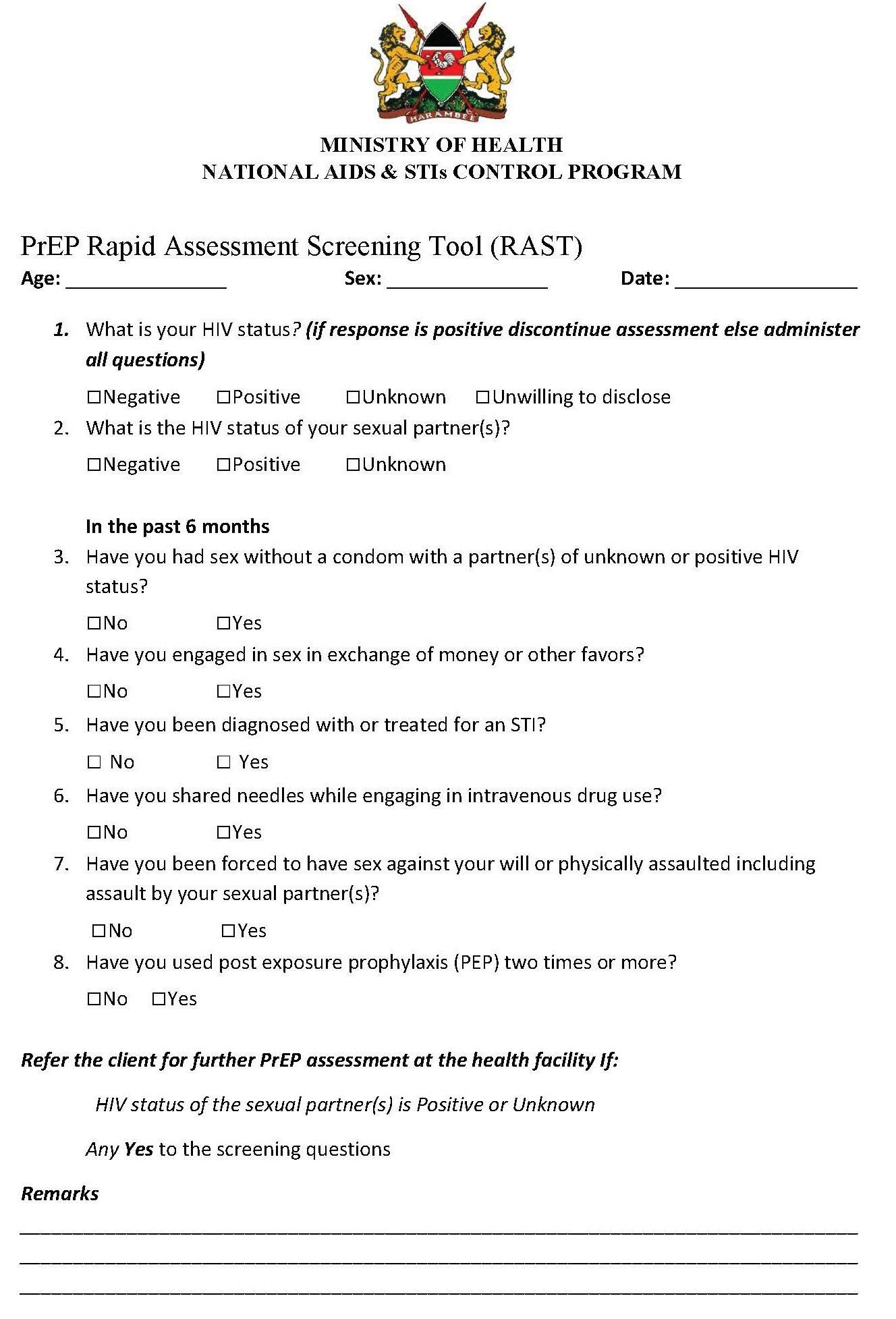

Supplement: Supplementary file 1 — Appendix S1: PrEP Rapid Assessment Screening Tool. [file JIA2-26-e26069-s001.docx]
